# Why harmonizing cohorts in sleep is a good idea and the labor of doing so?

**DOI:** 10.1093/sleep/zsae129

**Published:** 2024-06-14

**Authors:** Sarah Appleton, Jenny Theorell-Haglöw

**Affiliations:** Flinders Health and Medical Research Institute - Sleep Health (Adelaide Institute for Sleep Health), College of Medicine and Public Health, Flinders University, Bedford Park, SA, Australia; Department of Medical Sciences, Respiratory, Allergy and Sleep Research, Uppsala University, Uppsala, Sweden

Sleep researchers have worked tirelessly over the decades developing and validating questionnaires for the measurement of impaired sleep. Many of these are described in the book published in 2012 called *STOP, THAT, and 100 Other Sleep Scales* [1]. In addition, questions have been developed and administered to answer specific research questions that differ from these standard questionnaires and collectively the variety of items assessing aspects of sleep-related issues are vast. Cost, convenience, and consideration of participant impact necessitate the use of self-reported items in large-scale epidemiological studies. However, despite their clinical utility, it is arguable that this plethora of questionnaires for the assessment of various dimensions of sleep including quality, sleepiness, chronotype, and specific sleep disorders may also, to some degree, have hindered the advance of sleep epidemiology.

In as much as well-being is not just the absence of illness, sleep health is not just the absence of sleep disorders and their manifestations. A decade ago, Buysse et al. [2] recognized the importance of dimensions of sleep health beyond sleep duration as having independent impacts on health when they proposed the SATED tool. This assesses five dimensions associated with health outcomes, including subjective Satisfaction with sleep, along with four quantitative measures of Alertness during waking hours, Timing of sleep, Efficiency, and Duration of sleep. Sleep regularity was subsequently added to these sleep dimensions. Since then, a number of studies have examined the relationship of multidimensional sleep health with health outcomes including mental health [3–7], health status [8], cardiovascular disease [9, 10] and mortality [11], and report, perhaps not surprisingly, that higher numbers of healthy sleep behaviors are protective of poor outcomes (or inversely, higher numbers of dimensions of poor sleep predict poor outcomes). Limitations of the evidence base for multidimensional sleep health exist, however. For instance, many studies investigating the relationship between multidimensional sleep health and depression/mental health are limited to samples of almost exclusively women [3–6]. Sleep health dimensions cluster but studies that have conducted factor analyses of SATED components show inconsistent results regarding which sleep dimensions load on which factors [7, 12, 13]. Conflicting findings may in part relate to the participant profiles in these studies in addition to the framing of questionnaire items. More recent work extends this by considering multidimensional sleep health that includes objective measures including polysomnographic variables [14] and actigraphy [15].

There is a risk that individual studies have low statistical power for rarer sleep behaviors or outcomes, lack the ability to investigate special populations, and have limited generalizability of findings. Therefore, pooling or harmonizing data can be of great importance in addressing these issues and furthermore for determining the reproducibility, and generalizability of results from individual studies. Harmonizing subjectively assessed data across cohorts; however, is not without difficulty due to variations in question framing including quantity, frequency, or severity and time frames for assessment of information. In addition to this, when pooling or harmonizing data there are also possible legal issues where local, national, and international policies regarding data sharing can affect processes.

We read with interest the latest issue of *SLEEP* the paper by Wallace et al. [16], describing their harmonization work within the Sleep Harmonization, Aggregation, and REplication Initiative (SHARE). This initiative aims to leverage multi-cohort data to examine the extent to which sleep predicts various health outcomes—including cognitive decline and dementia—in older adults. We would like to acknowledge the immense effort they have made. They have established a multi-phase framework to harmonize self-reported sleep data and have then applied this process to harmonize and produce a pooled multi-cohort sample of five US cohorts and additionally a separate but fully harmonized cohort from the Netherlands. The framework has a four-step process from compiling items, creating domains from the items, harmonizing the items (i.e. semantic [identical variable names and constructs] and syntactic harmonization [identical coding, scales]), and finally external evaluation of the harmonizability of the data.

The complexity of sleep and the variability in its measurement is evident from the 190 unique self-reported sleep items that were identified by Wallace and colleagues across the six cohort studies. From these, 15 primary conceptual domains were created including Regularity, Satisfaction, Alertness/Sleepiness, Timing, Continuity, Duration, Sleep Apnea, Insomnia, Restless Leg Syndrome, Circadian Preference, Sleep Disturbances, Sleep Medication, General Sleep Disorder, Sleep Environment, and Other. There was a major variation in the number of items that were included within each domain. For example, Alertness/Sleepiness included 55 items while Regularity included only three items. In what was no doubt an arduous task, three experts independently classified all 190 items using the semantic item content into a primary domain and/or subdomain. A 5-point harmonizability scale scored pairs of items ranging from 0 (not harmonizable) to 4 (fully harmonizable). Several sophisticated harmonization metrics (H scores) were also developed to quantify harmonizability. From these 15 domains, 14 harmonized items were created and then externally evaluated and showing moderate to high harmonizability for 13 of these.

Perhaps not surprisingly, quantitative sleep dimensions (“total sleep time,” “time in bed,” “sleep efficiency,” “bedtime,” “wake-up time”) showed high potential for harmonization across cohorts compared to more subjective dimensions of sleep. “Sleep quality,” “feeling overly sleepy during the day,” “difficulty staying asleep,” and “stopping breathing during sleep” showed moderate potential for harmonization, while “excessive daytime sleepiness” and “taking naps” showed even lower potential for harmonization. “Daytime problems due to sleepiness” had the lowest potential for harmonization which is consistent with different questionnaire items assessing different aspects of daytime sleepiness (e.g. the ESS addresses introspective sleepiness). Insomnia (“difficulty falling asleep”) and sleep apnea (“snoring”) domains also showed high potential for harmonization. The moderate harmonizability potential of sleep apnea items of snoring, and stopping breathing is a valuable finding; however, self-report of symptoms is likely to underestimate the prevalence of the disorder and efforts to harmonize polysomnography data are required. Having said that, accurate multiple-night objective assessment of sleep is difficult and costly, although improvements in wearables/nearables may address this. Similarly, objective measures of sleep disturbance/insomnia symptoms may also provide better risk stratification however subjective sleep measures/perceptions also capture important exposures and ideally would be harmonized in combination with “objective” measures.

The work undertaken by Wallace et al. [16] contributes to advances in the field by enhancing reproducibility and generalizability of findings for practice and policy. Their present focus is sleep and aging research; however, for general health promotion, there is also a need for robust evidence across cohort populations of all ages, and with sex and racial/ethnic diversity given that sleep may be more modifiable than behaviors (e.g. addictive behaviors and physical activity) and non-modifiable factors such as age. We therefore also need to harmonize diverse cohorts to further be able to study sleep and sleep-related issues, and a broad range of outcomes and sleep health disparities across the whole life span.

Sleep is a complex phenomenon, and consequently, its measurement is complex. The use of non-standardized questions across cohort studies may limit advances in sleep epidemiology and the use of standardized items will help us adequately quantify the multi-dimensionality of sleep, changes over time, and its associated health risks. In addition to the framework improving data sharing, standardization, harmonization, and aggregation of sleep variables across studies, Wallace et al. [16] also provide us with a valuable list of essential and recommended activities for sleep cohort investigators and data custodians, while also pointing to possible obstacles for the process. They provide us with a guide for effective sleep harmonization and as they also state, this provides a foundation for further methodological development. The future of sleep health promotion requires initiatives to promote the use of common data elements in new and existing cohort data collections and we look forward to the implementation of this and other data harmonization frameworks in the future.

## References

[1] Shahid A , Wilkinson K, Marcu S, Shapiro C STOP, THAT and One Hundred Other Sleep Scales. New York, NY: Springer; 2012.

[2] Buysse DJ. Sleep health: can we define it? Does it matter? Sleep. 2014;37(1):9–17. doi: 10.5665/sleep.3298 24470692 PMC3902880

[3] Furihata R , Hall M, Stone K, et al. for the Study of Osteoporotic Fractures Research Group. An aggregate measure of sleep health is associated with prevalent and incident clinically significant depression symptoms among community-dwelling older women. Sleep. 2016;40(3). doi:10.1093/sleep/zsw075 PMC6084764 28364417

[4] Furihata R , Saitoh K, Suzuki M, et al. A composite measure of sleep health is associated with symptoms of depression among Japanese female hospital nurses. Compr Psychiatry. 2020;97:152151. 31954287 10.1016/j.comppsych.2019.152151

[5] Bowman MA , Kline CE, Buysse DJ, et al. Longitudinal association between depressive symptoms and multidimensional sleep health: The SWAN sleep study. Ann Behav Med. 2021;55:641–652. doi: 10.1093/abm/kaaa107 33410460 PMC8240133

[6] DeSantis AS , Dubowitz T, Ghosh-Dastidar B, et al. A preliminary study of a composite sleep health score: associations with psychological distress, body mass index, and physical functioning in a low-income African American community. Sleep Health. 2019;5(5):514–520. doi: 10.1016/j.sleh.2019.05.001 31208939 PMC6801051

[7] Appleton SL , Melaku YA, Reynolds AC, Gill TK, de Batlle J, Adams RJ. Multidimensional sleep health is associated with mental well-being in Australian adults. J Sleep Res. 2022;31(2):e13477. doi: 10.1111/jsr.13477 34622511

[8] Dalmases M , Benítez ID, Mas A, et al. Assessing sleep health in a European population: Results of the Catalan Health Survey 2015. PLoS One. 2018;13(4):e0194495. doi: 10.1371/journal.pone.0194495 29668685 PMC5905963

[9] Fan M , Sun D, Zhou T, et al. Sleep patterns, genetic susceptibility, and incident cardiovascular disease: a prospective study of 385 292 UK biobank participants. Eur Heart J. 2020;41(11):1182–1189. doi: 10.1093/eurheartj/ehz849 31848595 PMC7071844

[10] Li X , Xue Q, Wang M, et al. Adherence to a healthy sleep pattern and incident heart failure: a prospective study of 408802 UK Biobank participants. Circulation. 2020;143(0):97–99. doi: 10.1161/circulationaha.120.050792 33190528 PMC7775332

[11] Wallace ML , Buysse DJ, Redline S, et al. Multidimensional sleep and mortality in older adults: a machine-learning comparison with other risk factors. J Gerontol A Biol Sci Med Sci. 2019;74(12):1903–1909. doi: 10.1093/gerona/glz044 30778527 PMC6853700

[12] Brandolim Becker N , Martins RIS, Jesus SN, Chiodelli R, Stephen Rieber M. Sleep health assessment: a scale validation. Psychiatry Res. 2018;259:51–55. doi: 10.1016/j.psychres.2017.10.014 29028524

[13] Ravyts SG , Dzierzewski JM, Perez E, Donovan EK, Dautovich ND. Sleep health as measured by RU SATED: a psychometric evaluation. Behav Sleep Med. 2021;19(1):48–56. doi: 10.1080/15402002.2019.1701474 31829724 PMC7289662

[14] Chung J , Goodman M, Huang T, et al. Multi-dimensional sleep and mortality: The Multi-Ethnic Study of Atherosclerosis. Sleep. 2023;46(9). 10.1093/sleep/zsad048 PMC10848217 37523657

[15] Wallace ML , Yu L, Buysse DJ, et al. Multidimensional sleep health domains in older men and women: an actigraphy factor analysis. Sleep. 2021;44(2). doi: 10.1093/sleep/zsaa181 PMC7879411 32918075

[16] Wallace ML , Redline S, Oryshkewych N, et al. Pioneering a multi-phase framework to harmonize self-reported sleep data across cohorts. Sleep. 2024;47(9):1–16. doi: 10.1093/sleep/zsae115 PMC11381567 38752786

